# A Pilot Study on the Correlation Between Psychometric Traits and *ADRA2A* rs1800544 Polymorphism in Fencing Athletes

**DOI:** 10.3390/life15040625

**Published:** 2025-04-08

**Authors:** Beste Tacal Aslan, Begüm Su Baltacıoğlu, Özlem Özge Yılmaz, Tolga Polat, Korkut Ulucan

**Affiliations:** 1 Department of Basic Medical Sciences, Faculty of Dentistry, Marmara University, 34854 Istanbul, Türkiye; ozlem.ozge@marmara.edu.tr (Ö.Ö.Y.); tolga.polat@marmara.edu.tr (T.P.); korkutulucan@hotmail.com (K.U.); 2 Department of Molecular Biology and Genetics, Faculty of Engineering and Natural Sciences, Uskudar University, 34662 Istanbul, Türkiye; baltacioglu.begumsu@gmail.com; 3 Faculty of Medicine and Surgery, Cattolica del Sacro Cuore University, 25121 Bresci, Italy; 4 Personalized Treatment Application and Research Center, Uskudar University, 34768 Istanbul, Türkiye

**Keywords:** fencing, genetics, polymorphism, psychology, sports

## Abstract

Background: The aim of our study is to determine the genotype distribution of *ADRA2A* rs1800544 gene polymorphism in fencing athletes and to compare the results with the sedentary control group. In addition, we intend to compare these genotype results with the Depression Anxiety Stress Scale (DASS) and Sports Injury Anxiety Scale (SIAS) applied to the fencing group. Methods: Buccal epithelial cells were collected from 14 volunteer fencing athletes and 22 sedentary individuals who participated in the study and DNA was isolated. *ADRA2A* rs1800544 polymorphism was analyzed using Real-Time PCR. Data were analyzed using SPSS 25.0 software. Results: In the study, *ADRA2A* rs1800544 polymorphism was examined and 43% CG genotype, 57% CC genotype was found in fencers, and GG genotype was not detected. In the sedentary control group, 36.4% CC genotype, 45.5% CG genotype, and 18.1% GG genotype were detected; in both groups, C allele was found at a higher percentage than G allele. Although there were differences in the percentages of genotypes in the study and control groups, no statistically significant difference was found. Conclusions: The results obtained in our study reveal genotype distributions in line with the literature on *ADRA2A* rs1800544 gene region. Since fencing is a sport that requires attention and concentration, the distribution of gene regions associated with attention deficit and hyperactivity disorder is important. The results of this study contribute to the understanding of genotype distributions in fencing athletes; however, given the study’s limitations, such as the small sample size, lack of psychological assessment methods, and absence of noradrenaline level measurement, further research is needed before these findings can be considered for the development of personalized training programs based on *ADRA2A* rs1800544 polymorphism.

## 1. Introduction

Athletic performance is defined as the whole of physical and mental performance of athletes in any sportive activity and can be developed both physically and mentally with regular training. Research studies in sports genetics aim to investigate genes that impact athletic abilities, understand how they work, and analyse their role in determining an individual’s athletic potential [1]. The data from the Human Genome Project allow us to pinpoint the genes that influence athletic performance [2]. Factors like stress, anxiety, and aggression play a significant role in determining individuals’ performance in sports [3]. Identifying the genetic tendencies of athletes in high-pressure situations or in competitive sports helps them receive psychological support early on and can enhance their chances of succeeding in sports [4]. Recent research suggests that psychological factors such as anxiety and depression have significant effects on sports performance. In particular, it has been reported that high levels of anxiety may slow down information processing processes and affect performance negatively, but in some cases it may have positive effects on simple information processing tasks. These findings emphasize the importance of the relationship between athletes’ psychological state and performance [5].

In every sport, every athlete is at risk of injury to varying degrees over time. Psychological states, as well as physical strategies, also play a role in preventing injuries. Objective evidence indicates that psychological interventions that can significantly reduce the risk of injury involve addressing the mental factors that contribute to injury susceptibility and recovery. Particularly, methods for managing stress, including breathing exercises, cognitive restructuring, mental resilience training, mindfulness, and motor imagery, have been proven to be very effective [6]. Athletes may exhibit uncontrolled use of force, aggression, and risky behaviour in situations where they experience high levels of anxiety and depression. The psychological states of athletes should be analysed in more detail [7]. As well as the necessity of displaying a high level of physical performance throughout the competition, fencing athletes should also consider psychological variables such as attention, concentration, and motivation [8]. Fencing requires the sequential development of many mental strategies and the implementation of practices against ever-changing tactical moves in the process. Fencing athletes need to quickly evaluate their opponents and make rapid tactical and technical adaptations. Considering all of this, fencing largely encompasses the features of a mental game.

On the molecular level, adrenergic receptors—classified into alpha (ADRA) and beta (ADRB) groups—play vital roles in the regulation of the sympathetic nervous system. The ADRA2A gene, located on chromosome 10q24-q36, encodes the α2A-adrenergic receptor, which modulates physiological responses through its interaction with norepinephrine (noradrenaline) [9,10]. This receptor is part of the G protein-coupled receptor superfamily and specifically functions by inhibiting adenylyl cyclase activity. Animal studies have demonstrated that blocking ADRA2A receptors in the prefrontal cortex can result in notable cognitive and behavioral impairments [11], and disruptions in this system have been associated with neurodevelopmental conditions such as Attention-Deficit/Hyperactivity Disorder (ADHD). Research has indicated that *ADRA2A* may be a gene associated with ADHD [12]. Studies have shown that, in particular, rs1800544 (C-1291G) polymorphism in the promoter region of the *ADRA2A* gene has been linked to various traits, such as athletic endurance, body fat distribution, glucose and lipid metabolism, high blood pressure, salt excretion [13].

In this research, we analysed the genotype distribution of *ADRA2A* rs1800544 gene polymorphism in fencers and a control group of 22 sedentary individuals. Our aim was to explore whether this gene variant contributes to the psychological robustness and mental performance traits that are vital for success in high-cognitive demand sports such as fencing. We hypothesized that the genotype distribution of *ADRA2A* rs1800544 differs significantly between fencing athletes and sedentary individuals, suggesting a genetic predisposition for competitive success in sports requiring quick decision-making and high mental agility.

## 2. Materials and Methods

### 2.1. Study Group

In this pilot study, the study cohort was made up of 14 national fencers (female: 5, male: 9) who volunteered and 22 individuals (female: 11, male: 11) who lead sedentary lifestyles. The research protocol received approval from the Ethics Committee of Uskudar University (61351342/30 December 2022) and was conducted following the guidelines set out in the Declaration of Helsinki II. All fencers were informed about the experimental steps and ethical results, and the consent form was signed. Fencers trained at least 5 days a week, each session lasting at least 2 h. They performed two individual technical and strength/coordination exercises per week, as well as three days of technical and tactical work. In addition, in order to maintain their high mental condition, athletes are mentally conditioned by being exposed to artificial stress conditions in a controlled manner and are provided with regular meetings with a sports psychologist.

Eligibility criteria for study group:

Must be an active fencing athlete competing at the national or international level. Participants must be between 18 and 35 years old to control for age-related physiological and psychological variations. Athletes must have a minimum of 3 years of professional fencing experience and be actively engaged in structured training programs. Participants must have no prior diagnosis of severe psychiatric disorders and must not have been diagnosed with severe mental health conditions such as schizophrenia, bipolar disorder, or major depressive disorder that require clinical intervention. Athletes must provide written informed consent and be willing to complete all psychometric assessments (DASS and SIAS) and genetic testing. In the study, DNA samples were obtained by collecting buccal epithelial cells with the help of swabs from participants.

### 2.2. Depression Stress and Anxiety Scale (DASS)

The DASS was developed by Lovibond (1995) and consists of a total of 42 items, 14 of which belong to the depression, 14 to anxiety, and 14 to stress dimensions. High scores from each of the depression, anxiety, and stress dimensions reveal that the individual has a relevant problem [14]. The total scores of the scale, which does not contain any reverse items, vary between 0 and 42 for each subscale. The validity and reliability study of the scale was conducted by Akın and Çetin in 2007 [15]. Since fencing requires quick decision-making, precision, and mental resilience, fencers may experience heightened stress and anxiety during competition. The DASS scores will help quantify these psychological traits, allowing researchers to compare stress and anxiety levels among athletes with different *ADRA2A* rs1800544 genotypes.

### 2.3. Sport Injury Anxiety Scale (SIAS)

The SIAS was developed to assess the degree of anxiety experienced by athletes following an injury. This measurement tool was created by Rex and Metzler in 2016 [16] and later translated into Turkish by Caz and colleagues. In 2019, there are 6 sub-factors comprised of 19 items. These subcategories consist of fear of losing skills, fear of appearing fragile, fear of experiencing pain, fear of letdown, fear of losing social connections, and fear of re-injury [17].

### 2.4. DNA Isolation

Epithelial cells were collected from participants using sterile swabs. While buccal swabs offer a non-invasive and practical approach, they may yield lower DNA quality and quantity compared to blood samples, which could be considered a methodological limitation. A commercially available DNA isolation kit (Invitrogen; Thermo Fisher Scientific, Inc., Waltham, MA, USA) was used according to the manufacturer’s protocol. A swab was immersed in and removed from the ependorf containing PBS and the cells on the swab were transferred to the ependorf. Then, 200 µL of lysis buffer, 20 µL of proteinase K, and 5 µL of RNAse were added and pipetted. Immediately after this step, 200 µL of ethanol was added and was centrifuged at maximum speed for 1 min. Then, 500 µL of Wash Buffer 1 was added and was centrifuged. Wash Buffer 2 was centrifuged just like in the previous step. The filter tube was then transferred to a new 2 mL collection tube. After adding the buffer, it was centrifuged at maximum speed for one minute. The filter tube was discarded and a DNA sample of 125 µL remaining in the eppendorf was obtained. The concentration of the isolates was measured, an average of 20 ng of DNA was isolated from each sample (The Invitrogen Qubit 4 Fluorometer, Thermo Fisher Scientific, Inc., Waltham, MA, USA).

### 2.5. Genotyping

**Figure 1:**
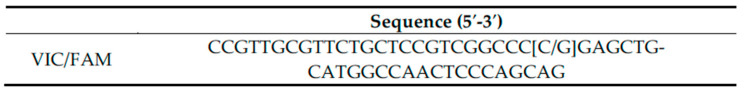
Probe sequence of *ADRA2A* rs1800544 polymorphism in Real-Time PCR.

*ADRA2A* rs1800544 polymorphism was performed using real time polymerase chain reaction (Real-Time PCR) on a StepOnePlus (Thermo Fisher Scientific, Inc.) using the TaqMan Genotyping test according to the manufacturer’s protocol (cat. No. 4362691; Thermo Fisher Scientific, Inc.) (Figure 1). PCR conditions were 60 °C for 30 s and 95 °C for 10 min, followed by 40 cycles at 95 °C for 15 s and 1 min at 60 °C. Finally, 60 °C was applied for 30 s for the post PCR reading.

### 2.6. Statistical Analysis

All data were analysed using SPSS 25.0 for Windows (SPSS Inc., Chicago, IL, USA). Statistical analysis was performed using chi-square test. Values less than *p* < 0.05 were considered significant. Descriptive data regarding socio-demographic information of participants are provided in the form of frequency tables. When examining the study data for normality assumptions, the Shapiro–Wilk (Kolmogorov–Smirnov) value was determined to be *p* < 0.05. Therefore, a Spearman correlation analysis, which is a non-parametric test, was conducted to examine the relationship between the scale and the survey scores. In addition, the non-parametric Mann–Whitney U test and Kruskal–Wallis H test were used to determine whether there were significant differences between the scale/test and the socio-demographic data of the participants. *p* < 0.05 was considered statistically significant.

## 3. Results

In the study, *ADRA2A* rs1800544 polymorphism was examined and according to the results, 6 (43%) of 14 fencers were found to have CG genotypes and 8 (57%) had CC genotypes. There was no fencer with the GG genotype for the *ADRA2A* rs1800544 polymorphisms. In the control group consisting of sedentary individuals, 8 (36.4%) had the CC genotype, 10 (45.5%) had the CG genotype, and 4 (18.1%) had the GG genotype. When allelic distributions were examined in both the fencing and control groups, the C allele was found to be higher in percentage compared to the G allele (Table 1).

The scale applied to only 14 fencing athletes and participant information are given starting from Table 2.

Descriptive statistics regarding the results obtained from the scale and sub-dimension scores are given in Table 3.

The scale applied to only 22 control participant information is given starting from Table 3.

Descriptive statistics regarding the results obtained from the scale and sub-dimension scores are given in Table 4.

The DASS Total score ranges from 2 to 45, with a mean of 20.29 (SD = 13.54), indicating moderate variability in overall psychological distress among fencers (Table 4). Among the subscales, Depression scores are relatively low (Mean = 3.29, SD = 4.08), suggesting that most athletes experience minimal depressive symptoms. Anxiety has a mean of 7.29 (SD = 4.71), indicating moderate anxiety levels among fencers. Stress shows the highest subscale mean (9.71, SD = 7.45), highlighting that stress may be a more prominent psychological challenge in fencing compared to depression or anxiety.

The SIAS Total score ranges from 27 to 64, with a mean of 41.64 (SD = 10.03), suggesting moderate to high levels of injury-related anxiety. Among the subscales: Re-Injury Anxiety has the highest mean (11.64, SD = 4.29), indicating that fear of re-injury is a major concern for fencers. Anxiety of Suffering (Mean = 9.43, SD = 2.38) also scores relatively high, implying that fencers may worry about pain and suffering due to injuries. The Anxiety of Losing Talent (Mean = 6.86, SD = 2.32) suggests that athletes are moderately concerned about injury affecting their skills. Other subscales, such as Anxiety of Disappointment (Mean = 5.21, SD = 3.19) and Anxiety of Losing Social Support (Mean = 4.36, SD = 2.44), have lower means, indicating that these concerns are less significant compared to re-injury anxiety.

The scores of the participants in Table 5 are compared based on gender for both the scale and subscale measurements. A significant statistical variation was observed in the stress score, a subscale of the DASS, based on gender. The stress score was discovered to be greater in females than in males (Z =−2.614, *p* = 0.009).

Table 6 compares participants’ scale and subscale scores regarding fear of injury. A statistically significant difference was found between the scores for fear of loss of social support. The scores for fear of loss of social support were higher in those who feared injury than those who did not (Z = −2.008 *p* = 0.045).

Table 7 compares the scale and subscale scores of participants based on the results of *ADRA2A* rs1800544 genotype. A statistically significant difference was found between the anxiety of being poor scores and *ADRA2A* rs1800544 genotype. Fear of being perceived as weak scores, one of the subscales of sports injury fear, were higher in the CG genotype than in the CC genotype (Z = −2.260, *p* = 0.024).

## 4. Discussion

Our primary objective was to investigate if athletes are genetically predisposed to attention and concentration disorders, with the aim of providing the information to prepare personalized training programs to address this predisposition proactively. Our findings emphasize that genetic and environmental factors should be considered together when evaluating the possible effects of *ADRA2A* rs1800544 polymorphism on psychological resilience in athletes. Studying the mechanism of action of genetic factors on sports and athletes contributes greatly to the regulation of sports performance [18]. In this context, in our study, we tested 14 fencers for the *ADRA2A* rs1800544 polymorphism. In our cohort, the CG genotype was more common than other genotypes, and the C allele was more common than the G allele. In this study, we did not encounter fencers with the GG genotype. Based on these results and the relationship between the homozygous G allele and inattention scores, our study suggests that *ADRA2A* is consistent with studies on the rs1800544 gene region [19]. Because fencing is a sport that requires a very high level of attention and concentration, it was observed that the CC genotype was more common than the CG genotype, while the GG genotype was not detected. However, given the limited sample size and the lack of a statistically significant difference in genotype frequencies, further studies with larger cohorts are needed to confirm this trend.

We compared the athletes’ psychological scale and subscale scores in terms of fear of injury. “The Anxiety of Losing Social Support” subscale score was statistically significantly different. We evaluated this result as indicating that the athletes who fear injury tend to have higher anxiety about losing social support compared to those who do not fear injury. This finding also suggests that the psychological impact of fear of injury extends beyond performance-related stress and into concerns about social acceptance and support within their environment.

A significant statistical variation was detected in the stress score between genders, females showed greater stress. Psychological, physiological, social factors, hormonal fluctuations (cortisol, estrogen, etc.) could be the reason for higher stress in females. In addition, females tend to have higher emotional reactivity and are more prone to performance anxiety, which can amplify stress in competitive environments.

The association between disorders of the central noradrenergic system and the *ADRA2A* gene is supported by previous studies [12]. Mutations in this gene may affect the psychological performance of athletes. Researchers found that individuals with the homozygous G allele had the highest scores for ADHD, particularly in terms of inattention [19]. Studies examining the effect of genetic polymorphisms on individuals’ psychological states provide important data to understand this relationship. In a study on the relationship between the rs6295 polymorphism of the *HTR1A* (5-hydroxytryptamine receptor 1A) gene and personality traits, significant differences were observed between mixed martial arts athletes and a control group. The study revealed that the rs6295 genotype was particularly effective on personality dimensions such as novelty seeking and harm avoidance, and individuals with the GG genotype had higher self-management scores and lower harm-avoidance scores. These results suggest that genetic factors may provide a basis for superior performance in combat sports [20].

There have been limited studies examining how the *ADRA2A* rs1800544 polymorphism impacts psychological performance in sports. According to a study looking at different parameters related to the rs1800544 gene region, Kambouris et al. (2012) found that among the parameters affecting athletes is energy expenditure, which affects biological processes such as lipolysis and thermogenesis, thereby leading to disruption of lipolysis control and fat accumulation [21]. At the same time, a study by Wolfarth et al. (2000) showed a role for adrenergic receptors in regulating adipose tissue lipolysis, a key step in the energy requirements of endurance training and examined possible associations with endurance performance [22]. A study conducted in 2018 by Eken et al., yielded similar findings, showing that Turkish kickboxing athletes predominantly possessed the CC genotype and the C allele of the *ADRA2A* rs1800544 polymorphism compared to other genotypes and alleles [4].

Only a small number of studies exist on the relationship between scales and gene polymorphisms in the available literature. In 2018, Ünal and colleagues conducted research on 121 children aged 6 to 18 with ADHD, focusing on the *ADRA2A* rs1800544 and catechol-O-methyltransferase (*COMT*) rs4680 genetic variations. The research examined the cognitive consequences on the brain. The Continuous Performance Test and Trail Making Test were used to evaluate performance. The symptoms and severity of ADHD were evaluated with the Conners’ Parent and Teacher Rating Scale. Ünal et al., (2018) found no connection between the *ADRA2A* rs1800544 and *COMT* rs4680 gene variations and particular neuropsychological characteristics based on the study results [23]. Zhang and Sun (2021) examined the likelihood of experiencing motion sickness based on the *ADRA2A* rs1800544 and *HTR3B* rs3758987 genetic variations using the Motion Sickness Susceptibility Questionnaires. The research discovered that individuals with the *ADRA2A* rs1800544 GG genotype were more likely to experience motion sickness, and this genetic variation heightened the likelihood of vomiting related to motion sickness [24]. In 2023, a study conducted by Tiring and Güloğlu focused on examining the emotional responses of electronic sports players. In the study, 136 e-sports players took part, with 33 females and 103 males, ranging in age from 15 to 45 years old. The study found that a significant portion of e-athletes experienced depression (20.6%), anxiety (14.7%), and stress symptoms (18.4%) [25].

Şen’s master’s thesis (2015) aimed to compare the levels of depression, anxiety, and stress in male students who played soccer professionally and those who did not. The results showed that depression, anxiety, and stress levels were higher in non-sports students than in those who played soccer professionally [26].

A study by Ekin and Bülbül (2020) investigated badminton players’ anxiety levels related to sports injuries according to SIAS. Male participants were found to have higher levels of anxiety than female participants, both in the total scale score and subscales of fear of perceived weakness and loss of social support. Furthermore, as physical activity increased, so did the level of anxiety about loss of subdivision of abilities. Study results showed male participants’ anxiety levels being higher than female participants [27].

A total of 305 participants, 144 female and 161 male, participating in individual and team sports, participated in a study that investigated the fear level of sports injuries among athletes based on various variables. The results showed that athletes participating in team sports had more anxiety in terms of pain and fear of re-injury compared to individual athletes. This increased anxiety is related to the desire of team sports athletes to prove themselves to their teammates and fear of losing one’s position. These results provide important evidence on how the social dynamics of team sports compared to individual sports can affect anxiety levels [28].

The psychological condition of fencing athletes plays a crucial role in their success, as mental resilience and focus are key components of peak performance in this highly strategic sport. Fencing demands rapid decision-making, acute awareness, and the ability to stay calm under pressure, all of which are heavily influenced by an athlete’s mental state. A strong psychological framework helps fencers manage stress, maintain concentration, and recover from setbacks quickly [29]. Moreover, the ability to visualize strategies and maintain a positive mindset can significantly enhance their tactical execution and adaptability during bouts. Ultimately, a well-developed psychological approach not only supports an athlete’s performance but also contributes to their overall well-being and longevity in sport [27].

## 5. Conclusions

In this pilot study, we focused on the *ADRA2A* gene variant to find an association between gene variation and the psychological endurance of fencers. The literature contains numerous studies that focus on scales used in sports. Nevertheless, only a small number of studies examined the relationship between the results of psychological trait questionnaires and the evaluation of genetic background. We assumed that the CC genotype would be higher in percentage in the fencing athletes’ group. There was no statistically significant difference in our study between the sedentary and athlete groups. If a significant correlation between *ADRA2A* rs1800544 polymorphism and psychometric traits has been found, fencing coaches and sports psychologists could develop personalized training programs tailored to the athlete’s genetic predisposition. By examining both the physical traits and genetic factors, we can forecast the potential abilities of individuals in various areas. However, it’s clear that psychological traits are influenced by multiple genes. Therefore, it is not solely reliant on a single gene variant. We ought to adopt a well-rounded perspective. Each individual should be considered as a unique case, taking into account both environmental and genetic factors as much as possible. Evaluating personality requires more than simply examining a single gene variant. Future studies should expand sample sizes to include athletes from various sports disciplines and different competitive levels to validate the findings across a broader population. Also, with the larger group study results according to the genetic background, nutritional supplements or pharmacological approaches targeting adrenergic pathways can optimize psychological resilience and athletic performance.

Considering all this information, our study has certain limitations. The limitations of our study comprise insufficient number of athletes, the lack of psychological assessment techniques, the use of the buccal swab method for DNA collection instead of blood samples, and absence of noradrenalin level measurement. However, we believe that our study will shed light on future sports genetics research and offer a new viewpoint. Our findings provide insight into the influence of genetic factors on athletic performance and represent an important step towards the development of individualized training programs according to psychological parameters. These results will help to better understand the role of genetics in athletic performance and guide future research in this field.

## Figures and Tables

**Table 1:** Genotype and allelic distribution of *ADRA2A* rs1800544 polymorphism between study and control groups.

	*ADRA2A* Genotype			*p* Value	Allele Distribution		*p* Value
	CC	CG	GG		C	G	
Study Group (*n* = 14)	8	6	-	0.184	22	6	0.087
Percentage	57.0%	43.0%	0.0%		79.0%	21.0%	
Control Group (*n* = 22)	8	10	4		26	18	
Percentage	36.4%	45.5%	18.1%		59.1%	40.9%	

Significance was evaluated as at least *p* < 0.05. The control group was analyzed using the χ2 test for comparison.

**Table 2:** Sociodemographic Data of Athletes.

Demographic Variables		*n*	%
Sex	Female	5	35.7
	Male	9	64.3
Body Mass Index	Healthy Weight	12	85.7
	Overweight	2	14.3
Siblings	No siblings	2	14.3
	1 siblings	10	71.4
	2 siblings	2	14.3
Training Per Week	1–3 times	5	35.7
	4 times and more	9	64.3
Fear of not Growing Tall	Yes	3	21.4
	No	11	78.6
Fear of Injury	Yes	5	35.7
	No	9	64.3
Chronic Disorder	Yes	4	28.6
	No	10	71.4
Drug Use	Yes	3	21.4
	No	11	78.6

**Table 3:** Sociodemographic Data of Control Group.

Demographic Variables		*n*	%
Sex	Female	10	45.5
	Male	12	54.5
Body Mass Index	Healthy Weight	15	68.2
	Overweight	7	31.8
Siblings	No siblings	5	22.7
	1 siblings	10	45.5
	2 siblings	7	31.8
Fear of not Growing Tall	Yes	8	36.4
	No	14	63.6
Fear of Injury	Yes	9	40.9
	No	13	59.1
Chronic Disorder	Yes	4	18.2
	No	18	81.8
Drug Use	Yes	3	13.6
	No	19	86.4

**Table 4:** Descriptive Statistics of Athlete Scores from the Scale and Subscales.

	Min	Max	Mean	Std. Deviation
**DASS Total**	2	45	20.29	13.54
Depression	0	15	3.29	4.08
Anxiety	1	17	7.29	4.71
Stress	1	29	9.71	7.45
**SIAS Total**	27	64	41.64	10.03
The Anxiety of Losing Talent	3	11	6.86	2.32
The Anxiety of Being Poor	3	8	4.14	1.79
Anxiety of Suffering	4	13	9.43	2.38
Anxiety of Disappointment	3	12	5.21	3.19
The Anxiety of Losing Social Support	3	10	4.36	2.44
Re-Injury Anxiety	4	20	11.64	4.29

**Table 5:** Comparison of Athlete’s Scale and Subscale Scores in Terms of Gender.

Variables	Mean ± SD	Z	*p*
**DASS Total**	F: 28.40 ± 12.56	−1.804	0.071
	M: 15.78 ± 12.46		
Depression	F: 3.20 ± 3.19	−0.543	0.587
	M: 3.33 ± 4.69		
Anxiety	F: 8.80 ± 4.49	−1.073	0.283
	M: 6.44 ± 4.88		
Stress	F: 16.40 ± 8.11	−2.614	**0.009**
	M: 6.00 ± 3.71		
**SIAS Total**	F: 44.40 ± 5.59	−1.068	0.286
	M: 40.11 ± 11.85		
The Anxiety of Losing Talent	F: 6.80 ± 2.49	−0.269	0.788
	M: 6.89 ± 2.37		
The Anxiety of Being Poor	F: 4.80 ± 2.17	−1.245	0.213
	M: 3.78 ± 1.56		
Anxiety of Suffering	F: 10.00 ± 1.22	−0.605	0.545
	M: 9.11 ± 2.85		
Anxiety of Disappointment	F: 4.20 ± 1.79	−0.642	0.521
	M: 5.78 ± 3.73		
The Anxiety of Losing Social Support	F: 5.00 ± 3.08	−0.753	0.452
	M: 4.00 ± 2.12		
Re-Injury Anxiety	F: 13.60 ± 2.51	−1.542	0.123
	M: 10.56 ± 4.80		

Z = Mann–Whitney U Test; *p* < 0.05.

**Table 6:** Comparison of Athletes’ Scale and Subscale Scores in Terms of Fear of Injury.

Variables	Fear of Injury	Mean ± SD	Z	*p*
**DASS Total**	Yes	15.80 ± 3.56	−0.267	0.789
	No	22.78 ± 16.50		
Depression	Yes	2.60 ± 1.52	−0.407	0.684
	No	3.67 ± 5.05		
Anxiety	Yes	5.40 ± 2.61	−1.073	0.283
	No	8.33 ± 5.41		
Stress	Yes	7.80 ± 2.17	−0.067	0.947
	No	10.78 ± 9.18		
**SIAS Total**	Yes	46.40 ± 10.83	−1.001	0.317
	No	39.00 ± 9.10		
The Anxiety of Losing Talent	Yes	6.80 ± 1.92	−0.067	0.946
	No	6.89 ± 2.62		
The Anxiety of Being Poor	Yes	4.20 ± 1.64	−0.078	0.938
	No	4.11 ± 1.96		
Anxiety of Suffering	Yes	10.00 ± 2.00	−0.538	0.591
	No	9.11 ± 2.62		
Anxiety of Disappointment	Yes	7.00 ± 4.06	−1.142	0.253
	No	4.22 ± 2.28		
The Anxiety of Losing Social Support	Yes	6.20 ± 3.27	−2.008	**0.045**
	No	3.33 ± 1.00		
Re-Injury Anxiety	Yes	12.20 ± 2.39	−0.469	0.639
	No	11.33 ± 5.17		

Z = Mann Whitney U Test; *p* < 0.05.

**Table 7:** Comparison of Participants’ Scale and Subscale Scores in Terms of *ADRA2A* rs1800544 Genotype Results.

Variables	*ADRA2A* rs1800544 Genotyping Results	Mean ± SD	Z	*p*
**DASS Total**	CC	23.13 ± 15.17	−0.906	0.365
	CG	16.50 ± 11.17		
Depression	CC	3.88 ± 4.85	−0.394	0.693
	CG	2.50 ± 3.02		
Anxiety	CC	8.00 ± 4.93	−0.844	0.399
	CG	6.33 ± 4.68		
Stress	CC	11.25 ± 9.05	−0.584	0.559
	CG	7.67 ± 4.55		
**SIA Total**	CC	37.88 ± 5.79	−1.486	0.137
	CG	46.67 ± 12.71		
The Anxiety of Losing Talent	CC	7.63 ± 2.00	−1.431	0.152
	CG	5.83 ± 2.48		
The Anxiety of Being Poor	CC	3.13 ± 0.35	−2.260	**0.024**
	CG	5.50 ± 2.07		
Anxiety of Suffering	CC	9.50 ± 2.78	−0.456	0.648
	CG	9.33 ± 1.97		
Anxiety of Disappointment	CC	4.38 ± 2.39	−0.622	X”
	CG	6.33 ± 3.98		
The Anxiety of Losing Social Support	CC	3.38 ± 1.06	−1.620	0.105
	CG	5.67 ± 3.20		
Re-Injury Anxiety	CC	9.88 ± 3.68	−1.623	0.105
	CG	14.00 ± 4.15		

Z = Mann–Whitney U Test; *p* < 0.05.

## Data Availability

The data presented in this study are available on request from the corresponding author.
